# Associations of obesity with newly diagnosed and previously known atopic diseases in Chinese adults: a case-control study

**DOI:** 10.1038/srep43672

**Published:** 2017-03-02

**Authors:** Biao Xie, Zhiqiang Wang, Yupeng Wang, Meina Liu, Yongchen Wang

**Affiliations:** 1 Department of Biostatistics, Public Health College, Harbin Medical University, Harbin City, Heilongjiang Province, China; 2 School of Medicine, the University of Queensland, Room 817, Health Sciences Building, Royal Brisbane & Women’s Hospital, Herston QLD, Australia; 3 Department of General Medicine, The Second Affiliated Hospital, Harbin Medical University, Harbin City, Heilongjiang Province, China

## Abstract

To assess the associations of obesity with newly diagnosed and previously known atopic disorders in Chinese adults. 4,629 adults aged 18 years or older were recruited in Harbin, China. Among them, 1,114 were previously diagnosed atopic cases, 1,298 were newly diagnosed cases, and 2,217 non-atopic controls. Obesity and overweight are defined according to the criteria established by the Working Group on Obesity in China. The associations of obesity with known and newly diagnosed atopic disorders were assessed using logistic regressions. Obesity was significantly associated with known atopic disorders (adjusted OR = 2.41 (95% CI: 1.81, 3.22)). The association of obesity with newly diagnosed atopic cases was not as strong as that with known cases, and was not statistically significant (adjusted OR = 1.27 (95% CI: 0.94, 1.72)). The similar pattern was observed in different allergic diseases, gender and age stratifications. The association between overweight and atopic diseases were not significant. Obesity is strongly associated with previously diagnosed atopic cases but not so with newly diagnosed atopic cases in Chinese adults. It is likely that people with atopic disorders have a higher risk of developing obesity. Our findings are important for the management of atopic disorders and chronic disease prevention among atopic disease patients.

There is a current debate on the association between obesity and atopic diseases in adults. It is still not clear if the recent epidemic of obesity has contributed to the rise in the incidence of individuals with atopic diseases or the rising incidence of atopic diseases has contributed to the increase in obesity. Some studies showed a positive association^1^,^2^,^3^,^4^ while others showed no association between obesity and atopic diseases in adults^5^,^6^. To understand the associations of obesity with those atopic diseases has important public health implications to elucidate the causal link and the importance of weight control in preventing atopic diseases. Previous studies often mixed newly diagnosed atopic cases with previously existing symptomatic cases. It is critical to separate the association of obesity with newly diagnosed atopic diseases from that with previously diagnosed atopic disease to understand the time sequence of the two events. The prevalence of obesity has been increased rapidly worldwide, particularly in low and middle income countries like China^7^. The incidence of atopic diseases, including atopic dermatitis, allergic rhinitis and allergic asthma, has also increased in China recently^8^. Therefore, understanding the association between obesity and atopic disease is particularly important for preventing and managing those conditions for this population. In this study, we assessed the associations of obesity with newly atopic disease and with previously diagnosed atopic disorders in Chinese adults.

## Results

### Characteristics of cases and controls

Table 1 shows the characteristics of controls, new and known atopic cases. In males, new cases and controls were different in family history while known cases were different from controls in age, weight, marital status, alcohol drinking, exercise, education, family history and the prevalence of obesity. In females, new cases and controls were different in age, residential region and cigarette smoking while known cases were different from controls in age, BMI, residential region, marital status, alcohol drinking, education, family history and the prevalence of obesity.

### Prevalence of obesity among new and known cases and controls

**Figure 1:**
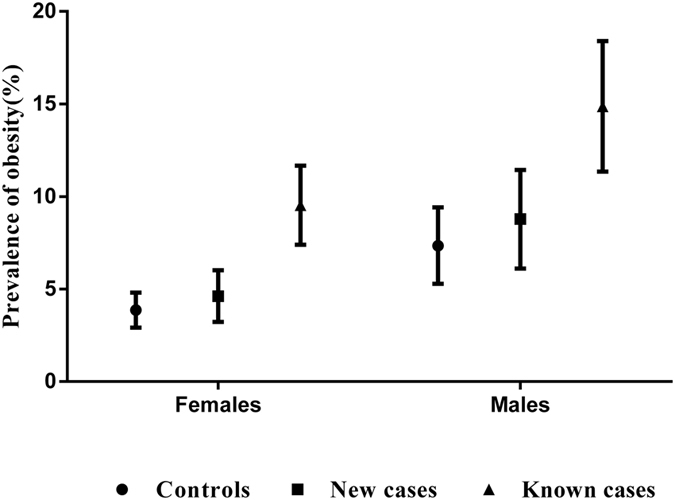
Prevalence of obesity among non-atopic controls, newly diagnosed and previously known atopic cases in Chinese adults.

Although the prevalence was lower in females than in males, the patterns of the relationships between obesity and atopic cases were the same in both genders. Both case groups had a higher prevalence of obesity than the control group. The previously known cases had a higher prevalence than the newly diagnosed case group, as shown in Fig. 1.

### Prevalence of obesity among atopic dermatitis, atopic rhinitis, atopic asthma, and multimorbidity of three atopic diseases

**Figure 2:**
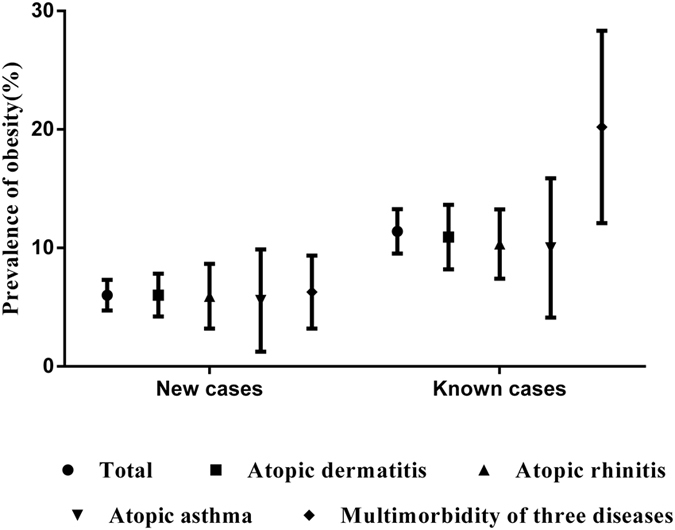
Prevalence of obesity among atopic dermatitis, atopic rhinitis, atopic asthma, and multimorbidity of three atopic diseases in Chinese adults.

Patients with different atopic diseases had different prevalence of obesity. Those with multimorbidity of three atopic diseases had a higher prevalence than the others, as shown in Fig. 2.

### Associations of obesity with newly diagnosed and previously known atopic diseases

Obesity was significantly associated with known atopic disease (crude OR = 2.54, 95% CI: 1.94, 3.32), as shown in Table 2. The association between obesity and newly diagnosed atopic disease was not significant (crude OR = 1.26, 95% CI: 0.93, 1.70). Adjusted for age, sex, education, cigarette smoking, alcohol drinking, marital status, family history of atopic diseases and physical exercise, the associations between obesity and known atopic cases remained strong and significant, adjusted OR = 2.41 (95% CI: 1.81, 3.22). The association between obesity and newly diagnosed atopic cases was weaker than that of obesity with known atopic cases, and remained non-significant, adjusted OR = 1.27 (95% CI: 0.94, 1.72). The similar patterns were observed in different allergic diseases, gender stratifications and age stratifications.

### ORs and 95% CIs for different atopic diseases in relation to BMI category

Table 3 presents the ORs and 95% CIs for different atopic diseases in relation to BMI category stratified by sex. Obesity was significantly associated with known atopic disease in both males (crude OR = 2.17, 95% CI: 1.42, 3.32) and in females (crude OR = 2.57, 95% CI: 1.80, 3.68). The association between obesity and newly diagnosed atopic disease was not significant (crude OR = 1.21, 95% CI: 0.76, 1.92 in males and OR = 1.17, 95% CI: 0.78, 1.76 in females). The associations between overweight and the two kinds of atopic diseases in both males were all not significant. Adjusted for age, sex, education, cigarette smoking, alcohol drinking, marital status, family history of atopic diseases and physical exercise, the associations between obesity and known atopic cases remained strong and significant, adjusted OR = 2.20 (95% CI: 1.38, 3.50) in males and adjusted OR = 2.67 (95% CI: 1.81, 3.91) in females. The association between obesity and newly diagnosed atopic cases was weaker than that of obesity with known atopic cases, and remained non-significant, adjusted OR = 1.29 (95% CI: 0.81, 2.06) in males and adjusted OR = 1.28 (95% CI: 0.84, 1.93) in females. The associations between overweight and the two kinds of atopic diseases in both males were still not significant.

## Discussion

In this study, we found that obesity was strongly and significantly associated with previously diagnosed atopic cases, and this association was different from that of obesity with newly diagnosed atopic cases in Chinese adults. The association of obesity with previously diagnosed existing atopic cases was much stronger than that with newly diagnosed cases, which implies it is likely that people with atopic disorders have higher risk of obesity.

In the current debate on the association between obesity and atopic diseases in adults, some studies support the presence of a positive association while others found no association between obesity and atopic diseases in adults^1^,^2^,^3^,^4^,^5^,^6^,^9^,^10^,^11^,^12^. In a study of 1,997 Canadian adults, Chen *et al*. found a significant association between obesity and atopic diseases with an adjusted odds ratio 1.33 (1.04, 1.71). In another study of 2,090 American adults, Silverberg *et al*. reported positive associations of obesity with atopic dermatitis and atopic asthma^2^. On the other hand, the data from Germany suggest no association between obesity and atopic diseases. The data from Australian adults showed no association between BMI and atopic diseases^13^. A multicentre cross-sectional survey of young adults in Europe showed that a positive association between high BMI and the risk of asthma attacks in women but there was no association between BMI and sensitization to any of allergens tested in the study^6^. Leung *et al*. showed that obesity was not associated with atopic diseases in Chinese children^14^. Little data are available from Chinese adults. In this study, we found that a significant association between obesity and the presence of a previously diagnosed atopic disease while a not significant association between obesity and newly diagnosed atopic diseases. Our findings are somewhat consistent with those of some previous studies of US adults^2^,^10^. Ma *et al*. reported no association between obesity and atopic diseases but an independent association of previously diagnosed asthma^10^. Silverberg *et al*. also found no association between obesity and atopic diseases but a significant association between obesity and symptomatic atopic dermatitis^2^. In this study, the atopic dermatitis, atopic rhinitis, atopic asthma, and multimorbidity of three atopic diseases were all significantly associated with obesity.

Having two sets of atopic case groups, newly diagnosed and previously diagnosed, is a unique feature in this study. A stronger association of obesity with previously diagnosed atopic cases than that with newly diagnosed atopic cases suggested that the duration of atopic cases might have played a role in the increased risk of obesity among the patients with atopic disorders. Although it is possible that the causal relationship could have existed in both directions: the presence of obesity increased the risk of atopic diseases and the presence of atopic diseases increased the risk of obesity, our data strongly support the latter. It is still not clear about why people with atopic diseases have a higher risk of obesity, but there are several possible explanations. Patients with previously diagnosed atopic diseases may be less physically active to avoid contact with allergens, and they are likely to store more energy than those without the condition. Also, some medications for treating atopic disorders may cause the patients to gain weight. Although we do not have evidence to support any of those explanations in the study population, our findings stress the importance of weight management and obesity prevention among atopic patients. Clinical guidelines for managing and treating atopic conditions should include the obesity prevention as a priority. We should also develop strategies, with appropriate choices of medications and advice on physical exercise and diet, specifically aiming to efficiently prevent obesity related chronic diseases among patient atopic disorders.

There are several strengths in this study. First, all cases and controls were confirmed by allergen-specific tests to sixteen common allergens in the region to minimise potential misclassification. Second, with two sets of different types of cases, we were able to obtain some evidence on time sequence between obesity and atopic diseases in Chinese adults.

However, there are some limitations in this study. As a case-control study, we were not able to identify exact times when either obesity or atopic diseases first occurred. Some newly diagnosed atopic cases might have had the condition for a quite a while but remained undiagnosed. Therefore, in the observed association between obesity and newly diagnosed atopic cases, obesity could have been either the cause or effect or both. Further cohort studies are needed to assess the detailed associations between obesity and atopic disorders, specifically aiming to assess if obesity increases the risk of atopic diseases in Chinese adults.

In conclusion, obesity is strongly associated with previously diagnosed atopic diseases but not so with newly diagnosed atopic disorders. Our data suggest that it is more likely that people with atopic disorders have a higher risk of obesity than that people with obesity have a higher risk of developing atopic disorders. Our findings are important for the management of atopic disorders and chronic disease prevention among atopic disease patients.

## Methods

### Study participants

In this case-control study, all atopic cases and controls were recruited from Harbin City in Heilongjiang Province of Northeast China. During March 2009 and December 2014, adult patients, aged 18 years or older, who visited the Department of Allergy of the First Affiliated Hospital of Harbin Medical University, were eligible for this study as potential cases. Potential controls were healthy adults who visited the same hospital for a health check-up during the same period. Potential cases and controls underwent allergen-specific IgE tests to sixteen allergens. We focused on the most common three atopic diseases, atopic dermatitis, asthma and rhinitis in this study. The current guidelines for allergic dermatitis^15^, rhinitis^16^ and asthma^17^ were used to diagnose them. The previously diagnosed atopic cases must meet the following criteria:

1. Had persistent positive IgE to at least one of the sixteen allergens in previous tests and in current test;
2. Had been diagnosed with at least one kind of atopic diseases mentioned above for more than one year and had also been diagnosed at this time.

Those meeting the following criteria were defined as newly diagnosed atopic cases:

1. Had positive IgE to at least one of the sixteen allergens in current test;
2. Diagnosed for the first time with at least one kind of atopic diseases mentioned above.

Multiple atopic cases were defined as those who had more than one kind of allergic diseases. Controls were those who had no previous history of atopic disease and were negative in all IgE concentrations. We conducted a questionnaire interview among 4,000 potential cases and 3,000 potential controls recruited. With the questionnaires with missing information or anomalous data excluded, we had 1,512 newly diagnosed cases, 1,297 previously diagnosed cases and 2,577 non-atopic controls. Questionnaires with logic errors and of specific populations (athletes and pregnant or breastfeeding women) were also excluded. Finally, our study included 1,298 newly diagnosed cases (664 atopic dermatitis, 287 atopic rhinitis, 108 atopic asthma, and 239 multimorbidity of three atopic diseases included), 1,114 previously diagnosed cases (504 atopic dermatitis, 416 atopic rhinitis, 100 atopic asthma, and 94 multimorbidity of three atopic diseases included) and 2,217 non-atopic controls.

### Measurements

#### Allergen-specific IgE testing

Allergen-specific IgE in serum concentrations to sixteen most common allergens in the region were measured using the AllergyScreen system (Mediwise Analytic GmbH, Germany) in all potential cases and controls. A positive atopic sensitisation was defined if the concentration of at least one of the allergen specific IgE was 0.35 kU/l or greater. The sixteen allergens were showed in Supplementary Table S1.

### Anthropometric measurements and obese

Body weight was measured to the nearest 0.1 kg using a calibrated standard scale with participants wearing light dress without shoes. Height was measured to the nearest 0.1 cm using a stadiometer. The physician who performed the anthropometric measurements did not know the nature of this study and the grouping of the study participants. Body mass index (BMI) was calculated as body weight (kg) divided by height (m) squared (kg/m^2^). All subjects were grouped into one of the following three groups, normal weight (BMI < 24), overweight (BMI ≥ 24 and BMI < 28) and obesity (BMI ≥ 28) according to the criteria established in 2003 by the Working Group on Obesity in China^18^.

### Demographic, family history and lifestyle factors

A structured questionnaire interview was conducted to collect data on the characteristics of study participants. Demographic factors included sex, age, education level, residential region and marital status. Lifestyle variables included cigarette smoking, alcohol drinking and physical exercise. The data on family history of related diseases such as atopic dermatitis, atopic rhinitis and atopic asthma were also collected. Age was calculated as the difference between the year of birth and the year of interview. Educational status was categorized into three levels: junior high school or lower, senior high school, and university or higher. Residential region was categorized into two groups: urban and rural. Marital status was also categorized into two groups: unmarried and married. Current smokers were defined on the basis of the World Health Organization criteria, as those who self-reported smoking every day for at least 6 months^19^. Regular alcohol drinkers were defined as drinking more than twice per week for at least one year. Classification of physical exercise was defined according to the criteria which take exercise intensity, time and frequency into consideration. The details are contained in the Supplementary Method. All subjects provided written informed consent to participate in this study. This project was approved by Harbin Medical University’s Ethical Review Committee. We confirm that all methods were performed in accordance with the relevant guidelines and regulations.

### Data analysis

The prevalence of obesity was calculated and compared among the three groups: controls, new cases and known cases. To assess the associations of obesity with new and known atopic disorders, we estimated odds ratios (OR) and their 95% confidence intervals using logistic regressions to adjust for potential confounding factors. We used stratification analysis to assess the associations of obesity with different types of known cases. Analyses were conducted for males and females separately, and all analyses were performed using SAS 9.1 (SAS Institute Inc., Cary, NC, USA)^20^.

## Additional Information

**How to cite this article**: Xie, B. *et al*. Associations of obesity with newly diagnosed and previously known atopic diseases in Chinese adults: a case-control study. *Sci. Rep.* **7**, 43672; doi: 10.1038/srep43672 (2017).

**Publisher's note:** Springer Nature remains neutral with regard to jurisdictional claims in published maps and institutional affiliations.

## Supplementary Material

Supplementary Information

## Figures and Tables

**Table 1:** Characteristics of new and known atopic cases and controls in Chinese adults.

	Control	New cases	P^1^	Known cases	P^1^
**Male**					
Number	613	433		390	
Age, years	35.84 (16.91)	34.34 (17.31)	0.1611	31.96 (14.91)	0.0002
Height, cm	167.40 (16.69)	168.10 (17.21)	0.4853	169.30 (15.57)	0.0616
Weight, kg	66.33 (17.93)	66.99 (19.72)	0.5766	69.18 (18.67)	0.0160
BMI, kg/m^2^	23.18 (4.38)	23.11 (4.56)	0.8166	23.66 (4.54)	0.0953
Rural, n (%)	203 (33.12)	154 (35.57)	0.4104	118 (30.26)	0.3440
Married, n (%)	377 (61.50)	255 (58.89)	0.3953	210 (53.85)	0.0165
Cigarette smoking, n (%)	223 (36.38)	175 (40.42)	0.1853	126 (32.31)	0.1870
Alcohol drinking, n (%)	216 (35.24)	149 (34.41)	0.7826	104 (26.67)	0.0045
Physical exercise, n (%)	240 (39.15)	172 (39.72)	0.8523	184 (47.18)	0.0121
Education					
Junior high school or lower	287 (46.82)	208 (48.04)	0.9250	151 (38.72)	0.0412
Senior high school	141 (23.00)	98 (22.63)		102 (26.15)	
University or higher	185 (30.18)	127 (29.33)		137 (35.13)	
Family history, n (%)	72 (11.75)	70 (16.17)	0.0398	176 (45.13)	<0.0001
Obese, n (%)	45 (7.34)	38 (8.78)	0.3977	58 (14.87)	0.0001
**Female**					
Number	1,604	865		724	
Age, years	38.18 (13.64)	36.26 (14.02)	0.0010	36.80 (14.52)	0.0305
Height, cm	160.80 (7.77)	160.20 (10.23)	0.1360	160.40 (9.67)	0.2405
Weight, kg	57.53 (10.34)	57.04 (11.67)	0.3051	58.18 (11.67)	0.1957
BMI, kg/m^2^	22.16 (3.57)	22.04 (3.62)	0.4136	22.53 (3.91)	0.0319
Rural, n (%)	565 (35.22)	257 (29.71)	0.0055	200 (27.62)	0.0003
Married, n (%)	1,254 (78.18)	652 (75.38)	0.1132	522 (72.10)	0.0014
Cigarette smoking, n (%)	138 (8.60)	53 (6.13)	0.0280	50 (6.91)	0.1641
Alcohol drinking, n (%)	93 (5.80)	58 (6.71)	0.3695	15 (2.07)	<0.0001
Physical exercise, n (%)	446 (27.81)	258 (29.83)	0.2886	206 (28.45)	0.7474
Education					
Junior high school or lower	679 (42.33)	348 (40.23)	0.1698	270 (37.29)	0.0022
Senior high school	448 (27.93)	228 (26.36)		186 (25.69)	
University or higher	477 (29.74)	289 (33.41)		268 (37.02)	
Family history, n (%)	223 (13.90)	135 (15.61)	0.2512	312 (43.09)	<0.0001
Obese, n (%)	62 (3.87)	40 (4.62)	0.3660	69 (9.53)	<0.0001

^1^P values comparing cases with controls.

**Table 2:** Association of obesity with newly diagnosed and previously known atopic cases in Chinese adults.

Cases		Obesity N (%)^a^	Crude	P	Adjusted^b^	P
			OR (95% CI)		OR (95% CI)	
Newly diagnosed	Male	433 (8.78)	1.21 (0.77–1.91)	0.3983	1.25 (0.79–1.97)	0.3339
	Female	865 (4.62)	1.21 (0.80–1.81)	0.3666	1.29 (0.85–1.94)	0.2270
	18–39	775 (4.90)	1.32 (0.85–2.05)	0.2223	1.33 (0.85–2.07)	0.2192
	40–59	466 (7.51)	1.30 (0.84–2.02)	0.2460	1.26 (0.81–1.97)	0.3120
	≥60	57 (8.77)	1.21 (0.39–3.78)	0.7464	1.38 (0.42–4.50)	0.5930
	Atopic dermatitis	664 (6.02)	1.26 (0.87–1.84)	0.2194	1.33 (0.91–1.95)	0.1356
	Atopic rhinitis	287 (5.92)	1.24 (0.73–2.10)	0.4208	1.27 (0.74–2.18)	0.3907
	Atopic asthma	108 (5.56)	1.16 (0.50–2.70)	0.7309	1.08 (0.46–2.54)	0.8587
	Multimorbidity of three atopic diseases	239 (6.28)	1.32 (0.76–2.31)	0.3279	1.24 (0.71–2.18)	0.4524
	Total	1,298 (6.01)	1.26 (0.93–1.70)	0.1303	1.27 (0.94–1.72)	0.1238
Previously known	Male	390 (14.87)	2.21 (1.46–3.33)	0.0002	2.06 (1.32–3.21)	0.0016
	Female	724 (9.53)	2.62 (1.84–3.74)	<0.0001	2.64 (1.80–3.86)	<0.0001
	18–39	690 (10.58)	3.02 (2.06–4.43)	<0.0001	2.70 (1.79–4.09)	<0.0001
	40–59	374 (10.96)	1.97 (1.29–3.02)	0.0018	1.89 (1.19–3.02)	0.0073
	≥ 60	50 (26)	4.41 (1.75–11.15)	0.0017	5.03 (1.94–12.99)	0.0009
	Atopic dermatitis	504 (10.91)	2.42 (1.72–3.40)	<0.0001	2.29 (1.59–3.29)	<0.0001
	Atopic rhinitis	416 (10.34)	2.27 (1.57–3.29)	<0.0001	2.37 (1.59–3.54)	<0.0001
	Atopic asthma	100 (10.00)	2.19 (1.11–4.33)	0.0240	2.32 (1.13–4.78)	0.0224
	Multimorbidity of three atopic diseases	94 (20.21)	5.00 (2.91–8.57)	<0.0001	5.24 (2.98–9.24)	<0.0001
	Total	1,114 (11.40)	2.54 (1.94–3.32)	<0.0001	2.41 (1.81–3.22)	<0.0001

^a^N, number of cases; percentage, prevalence of obesity among atopic cases.

^b^Adjusted for age, sex, education, cigarette smoking, alcohol drinking, marital status, family history of atopic diseases and physical exercise.

**Table 3:** ORs and 95% CIs for atopic diseases in relation to BMI.

	Control N (%)	Newly diagnosed N (%)	cOR^1^ (95% CI)	aOR^1^ (95% CI)	Previously known N (%)	cOR^1^ (95% CI)	aOR^1^ (95% CI)
**Male**							
Normal	357 (58.24)	249 (57.51)	1.00 (ref)	1.00 (ref)	212 (54.36)	1.00 (ref)	1.00 (ref)
Overweight	211 (34.42)	146 (33.72)	0.99 (0.76–1.29)	1.07 (0.81–1.42)	120 (30.77)	0.96 (0.72–1.27)	1.18 (0.86–1.62)
Obesity	45 (7.34)	38 (8.78)	1.21 (0.76–1.92)	1.29 (0.81–2.06)	58 (14.87)	2.17 (1.42–3.32)	2.20 (1.38–3.50)
**Female**							
Normal	1,179 (73.50)	649 (75.03)	1.00 (ref)	1.00 (ref)	510 (70.44)	1.00 (ref)	1.00 (ref)
Overweight	363 (22.63)	176 (20.35)	0.88 (0.72–1.08)	0.97 (0.78–1.20)	145 (20.03)	0.92 (0.74–1.15)	1.04 (0.83–1.32)
Obesity	62 (3.87)	40 (4.62)	1.17 (0.78–1.76)	1.28 (0.84–1.93)	69 (9.53)	2.57 (1.80–3.68)	2.67 (1.81–3.91)

c OR, crude odds ratio; CI, confidence interval. a OR, adjusted odds ratio, adjusted for age, sex, education, cigarette smoking, alcohol drinking, marital status, family history of atopic diseases and physical exercise. ^1^OR values comparing cases with controls.
