# Supplementary material for: Associations of obesity with newly diagnosed and previously known atopic diseases in Chinese adults: a case-control study
Source: Sci Rep. 2017 Mar 2;7:43672. doi: 10.1038/srep43672 (PMC5333111; doi:10.1038/srep43672)
Supplement: Supplementary Information [file srep43672-s1.doc]

**Associations of obesity with newly diagnosed and previously known atopic diseases in Chinese adults****: a case–control study**

Biao Xie^1^, Zhiqiang Wang^2^, Yupeng Wang^1^, Meina Liu^1^*, Yongchen Wang^3^*

^1^Department of Biostatistics, Public Health College, Harbin Medical University, Harbin City, Heilongjiang Province, China.

^2^School of Medicine, the University of Queensland, Room 817, Health Sciences Building, Royal Brisbane & Women’s Hospital, Herston QLD, Australia.

^3^Department of General Medicine, The Second Affiliated Hospital, Harbin Medical University, Harbin City, Heilongjiang Province, China.

*Correspondence author: Tel: +86-451-87502680 E-mail: [liumeina369@163.com](mailto:liumeina369@163.com)

*Correspondence author: Tel: +86-451-86605727 E-mail: yongchenwang@163.com

**Supplementary Method:**

Classification of physical exercise (PE) was defined according to the criteria:

We estimated PE condition of study participants using three variables of structured questionnaire, exercise intensity (EI), exercise time (ET) and exercise frequency (EF) ^1-4^.

EI of study participants were classified into three groups, mild, moderate and strenuous exercise according to their exercise types. Value 1, 2 and 3 were assigned to the three groups respectively ^5-8^. ET was measured by the hour. EF referred to how many times a week. We defined PE as

$$PE=\sum_{i=1}^{N} EI\times ET$$

Where$N$represented EF. If PE ≥ 2, PE was positive; PE was negative when PE < 2. Therefore, as for a subject jogging (moderate exercise) 15 minutes 5 times a week, his/her PE was positive because PE ≥ 2 (PE = 5× 0.25 × 2 = 2.5).

**References**

1. Schoenmakers, P., Reed, K., Van Der Woude, L., & Hettinga, F. J. High Intensity Interval Training in Handcycling: The Effects of a 7 Week Training Intervention in Able-bodied Men. *Front. Physiol.* **7,** 638 (2016).

2. Tikkanen-Dolenc, H. *et al*. Frequent and intensive physical activity reduces risk of cardiovascular events in type 1 diabetes. *Diabetologia* doi: 10.1007/s00125-016-4189-8 (2016).

3. Mayer, J. M. *et al*. Effect of Lumbar Progressive Resistance Exercise on Lumbar Muscular Strength and Core Muscular Endurance in Soldiers. *Mil. Med.* **181,** e1615- e1622 (2016).

4. King, A. C. *et al*. Preserving older adults' routine outdoor activities in contrasting neighborhood environments through a physical activity intervention. *Prev. Med.* **96,** 87-93 (2016).

5. Parks, C. M. & Manohar, M. Distribution of blood flow during moderate and strenuous exercise in ponies (Equus caballus). *Am. J. vet. Res*. **44,** 1861-1866 (1983).

6. Vijayaraghava, A., Doreswamy, V., Narasipur, O. S., Kunnavil, R. & Srinivasamurthy, N. Effect of yoga practice on levels of inflammatory markers after moderate and strenuous exercise. [*J. Clin. Diagn. Res*](https://www.ncbi.nlm.nih.gov/pubmed/?term=Effect+of+Yoga+Practice+on+Levels+of+Inflammatory+Markers+After+Moderate+and+Strenuous+Exercise)*.* **9,** CC08-12 (2015).

7. Wang, J. S. *et al*. Different effects of strenuous exercise and moderate exercise on platelet function in men. *Circulation* **90,** 2877-2885 (1994).

8. Duzova, H., Karakoc, Y., Emre, M. H., Dogan, Z. Y. & Kilinc, E. Effects of Acute Moderate and Strenuous Exercise Bouts on IL-17 Production and Inflammatory Response in Trained Rats. *J. Sports Sci. Med.* **8,** 219-224 (2009).

**Supplementary Table:**

**Supplementary Table S1.** Sixteen common allergens used in this study

| *Dermatophagoides pteronyssinus* | mould mixture ^a^ | fish | beef |
| --- | --- | --- | --- |
| egg white/egg yolk | German cockroach | crab | shrimp |
| common ragweed and mugwort | blue mussel | mutton | wheat |
| cat and dog fur | tree pollen mixture ^b^ | milk | Hop |

^a^ Mould mixture is composed of *Penicillium notatum, Cladosporium herbarum, Aspergillus fumigatus* and *Alternaria alternate.*

^b^ Tree pollen mixture is composed of *Robur, Elm, London Plane, Willow* and *cottonwood*.
